# Association Between Statin Use in the Intensive Care Unit and Delirium in Patients Receiving Mechanical Ventilation: A Cross‐Sectional Study

**DOI:** 10.1002/hsr2.71013

**Published:** 2025-07-21

**Authors:** Zhi Liu, Zhichao Zou, Zhe Li, Qihai Wan, Yi Yu

**Affiliations:** ^1^ Department of Anesthesiology The First Hospital of Hunan University of Chinese Medicine Changsha Hunan Province China; ^2^ Department of Intensive Care Medicine Juancheng County People's Hospital Heze Shandong China; ^3^ Department of Anesthesiology, West China Second University Hospital Sichuan University Chengdu Sichuan China; ^4^ Key Laboratory of Birth Defects and Related Diseases of Women and Children (Sichuan University) Ministry of Education Chengdu Sichuan China; ^5^ Department of Critical Care Medicine The Second Affiliated Hospital of Guangzhou University of Chinese Medicine Guangzhou Guangdong China

**Keywords:** delirium, ICU, mechanical ventilation, MIMIC‐IV, statins

## Abstract

**Background and Aims:**

Delirium is a temporary cognitive dysfunction caused by organic factors. It is characterized by impaired attention and cognitive abilities. This condition is associated with a high prevalence of misdiagnosis and considerable risks of disability and mortality, particularly in intensive care unit (ICU) patients undergoing mechanical ventilation (MV). This study aimed to investigate the impact of the use of statins on the occurrence of delirium in patients undergoing MV in the ICU by applying a comprehensive data set.

**Methods:**

The Medical Information Mart for Intensive Care‐IV (MIMIC‐IV) database was used for participant recruitment. The primary outcome was the prevalence of delirium. Secondary outcomes were the duration of ICU stay (DoIS) and duration of hospital stay (DoHS). Multivariable logistic regression and multivariate linear regression were employed to carry out statistical analyses. Propensity‐score matching (PSM) was utilized to enhance the robustness of findings. Statin use was determined based on patients' medication history before ICU admission. All the examinations and tests were conducted within 24 h after patients were admitted to the ICU.

**Results:**

The study comprised 18,146 participants with a mean age of 63.4 years and the prevalence of delirium was 7.6% (1381/18,146). According to multivariable logistic regression models, patients prescribed statins exhibited a 15% higher prevalence of delirium (odds ratio = 1.15, 95% confidence interval = 1.01–1.37, *p* < 0.05). Statin administration in the ICU correlated with a 4.03‐h decrease in ventilation time (*p* < 0.001). These results suggest that statin use may increase the risk of delirium in patients undergoing MV, even following stratification of statin use and PSM. Statins did not have a significant impact on DoIS, however, their use was associated with a longer DoHS.

**Conclusion:**

The findings of this retrospective cross‐sectional study indicate that statin use is linked to an increased risk of delirium in patients receiving MV in the ICU.

## 1 Introduction

Delirium is a reversible condition with a sudden onset. It is defined by impairments in cognition and consciousness, disturbances in the sleep–wake cycle, and fluctuations in psychomotor activity. Critically ill individuals are particularly susceptible to experiencing delirium, and 80% of patients requiring mechanical ventilation (MV) suffer delirium [1, 2]. These circumstances may result in supplementary diagnostic evaluations, medication administration, postponed removal of an endotracheal tube, prolonged durations in the intensive care unit (ICU) and hospital, increased susceptibility to nosocomial infections, and a heightened risk of hospital readmission [3]. Cognitive dysfunction produces unfavorable effects on the quality of life and generates augmented healthcare expenditures [4, 5]. Notwithstanding the high prevalence of delirium, few pharmacological options are available for mitigating its associated symptoms [6].

The etiology of delirium is not known, but its development can be influenced by neurobiological mechanisms (e.g., neuroinflammation, neurological dysfunction) as well as common triggers (e.g., hypoglycemia, impaired supply of oxygen to the brain) [7, 8]. Neuroinflammation, in concert with the linked processes of oxidative damage and apoptosis, constitutes a critical component of the pathophysiological basis of delirium. Consequently, patients in the ICU who experience delirium may develop long‐term cognitive deficits [9, 10]. Neuroinflammation is considered to be a significant contributor to delirium development. It has been suggested that administration of anti‐inflammatory drugs may reduce neuroinflammatory processes and alleviate delirium symptoms subsequently. However, further research is required to establish the efficacy and safety of such interventions.

The literature provides robust evidence supporting the notion that the main pharmacological effect of statins is anti‐inflammatory [11, 12, 13]. The PADIS 2018 guideline emphasizes a multi‐component approach for delirium prevention, yet the specific role of statins in achieving these prevention goals remains to be fully elucidated [14]. Statins are commonly used in the management of hypercholesterolemia associated with atherosclerotic cardiovascular disease. Statins have inhibitory effects on the secretion of pro‐inflammatory cytokines such as interleukin (IL)−2, interferon‐γ, IL‐6, and tumor necrosis factor‐α. These anti‐inflammatory properties may have a modulating impact on neuroinflammation by mitigating microglial activation and suppressing the production of pro‐inflammatory mediators [12, 15]. Investigations have demonstrated the advantageous impact of statins on cerebral vasospasm. Statins can facilitate the structural and functional reorganization of vascular endothelial cells to ultimately enhance cerebral vascular autoregulation and perfusion [16, 17].

The literature offers limited and inconclusive evidence on the effects of statins on delirium in patients requiring MV [1, 18, 19, 20, 21, 22]. This knowledge gap was addressed in a cross‐sectional study undertaken herein.

## 2 Methods

We included patients undergoing invasive MV, with or without previous statin use, in the Medical Information Mart for Intensive Care‐IV (MIMIC‐IV, version 2.2), which is a retrospective, single‐center database encompassing data from 2008 to 2019 in the USA [23]. The MIMIC‐IV database became publicly available after receiving IRB approval from two institutions. These are the Beth Israel Deaconess Medical Center in Boston, MA, USA (2001‐P‐001699/14) and the Massachusetts Institute of Technology, MA, USA (0403000206), and were granted a waiver of informed consent. In addition, since the research data does not come from the Second Affiliated Hospital of Guangzhou University of Chinese Medicine (Guangdong Provincial Hospital of Chinese Medicine), we have obtained an ethical waiver from the Ethics Committee of Guangdong Provincial Hospital of Chinese Medicine (AF/02−07.1/12.0). Written informed consent to participate in this study was provided by the participants' legal guardian/next of kin. Yi Yu obtained the necessary authorization (certificate ID: 6477678) to utilize the database. The present study was conducted in accordance with the Guidelines for Strengthening the Reporting of Observational Studies in Epidemiology [24].

### 2.1 Inclusion and Exclusion Criteria

The inclusion criteria were patients requiring invasive MV and aged ≥ 18 years. If a patient had multiple admissions to the ICU, only the initial admission was included. Patients with an ICU stay < 24 h were excluded. Furthermore, the study excluded cases of delirium occurring before the initiation of statin therapy.

### 2.2 Study Population and Data Extraction

We enrolled patients who underwent invasive MV. Delirium diagnosis was based on the Confusion Assessment Method for the ICU (CAM‐ICU) criteria [25]. The end point was reached when a patient first fulfilled the CAM criteria for delirium during their stay in the ICU Relevant patient characteristics, comorbidities, clinical‐severity scores, treatments (e.g., ventilation, dialysis, administration of vasoactive medication), vital signs and laboratory findings (ICU admission results within 24 h, or average of multiple results), as well as other data obtained upon hospital admission, were collected systematically.

### 2.3 Statin Use

Statin use was defined operationally as the presence of statin prescriptions recorded before the ICU admission within the MIMIC‐IV database. Per the inclusion criteria, the administration of statins should occur before the onset of delirium.

### 2.4 Covariates

In line with previous studies [26, 27, 28, 29], prognostically significant covariates associated specifically with delirium in patients undergoing invasive MV were included in our analyses.

The covariates considered in this study were: insurance status; marital status; ethnicity; age; sex; body mass index (BMI); hours of sedation and analgesia: Simplified Acute Physiology Score (SAPS) II; Sequential Organ Failure Assessment (SOFA) score; presence of sepsis; Charlson Comorbidity Index; arterial pH; arterial partial pressure of oxygen (PaO_2_); arterial partial pressure of carbon dioxide (PaCO_2_); ratio of PaO_2_‐to‐fraction of inspired oxygen (PaO_2_:FiO_2_); lactate level; white blood cell (WBC) count; platelet count; body temperature; heart rate; mean blood pressure; respiratory rate; oxygen saturation (SpO_2_); duration of ICU stay (DoIS); number of hours of MV; continuous renal replacement therapy (CRRT); duration of hospital stay (DoHS); use of vasoactive drugs; presence of myocardial infarction, congestive heart failure, cerebrovascular disease, chronic pulmonary disease, renal disease, malignant cancer, rheumatic disease, or diabetes mellitus; levels of hemoglobin, serum creatinine, blood urea nitrogen (BUN), glucose, sodium, and potassium.

### 2.5 Outcomes

The primary objective was the prevalence of delirium. The secondary objectives were DoIS and DoHS.

### 2.6 Statistical Analysis

Participants were separated into different groups based on age, sex, BMI, and comorbidities. We used analysis of variance or rank sum testing to evaluate differences in continuous variables. The characteristics of patients in different groups at baseline are presented as numbers and percentages for categorical data and mean ± standard deviation or median (interquartile range) for continuous data. This strategy allowed a comprehensive assessment of patient characteristics and helped to identify potential confounding factors. To compare the characteristics of the study population across different outcome groups, *χ* ^2^ or Fisher's exact tests were employed for categorical variables.

Due to the relatively low percentage of missing data (ranging between 0.3% and 4%) for height and weight, an imputation method was not utilized. Instead, the missing data in vital signs and laboratory parameters (which accounted for 5% of the total) were replaced with the median value. Multivariate logistic regression analysis was undertaken to assess the specific relationship between the use of statins and the occurrence of delirium. Subsequent analyses (which accounted for relevant covariates) encompassed subgroup analyses and analyses of interactions. Propensity‐score matching (PSM), a statistical technique for observational studies, reduces bias from confounding variables when comparing treatment and control groups. To enhance the robustness of findings, PSM was conducted using a 1:1 nearest neighbor‐matching algorithm and a caliper width of 0.02.

Statistical analyses were conducted using STATA version 17.0 (http://www.stata.com), R (http://www.R-project.org), and Free Statistics version 1.8. Multiple imputation was employed to account for missing values in logistic regression models. *p* < 0.05 (two‐tailed) was considered significant.

## 3 Results

### 3.1 Participants and Baseline Characteristics

**Figure 1:**
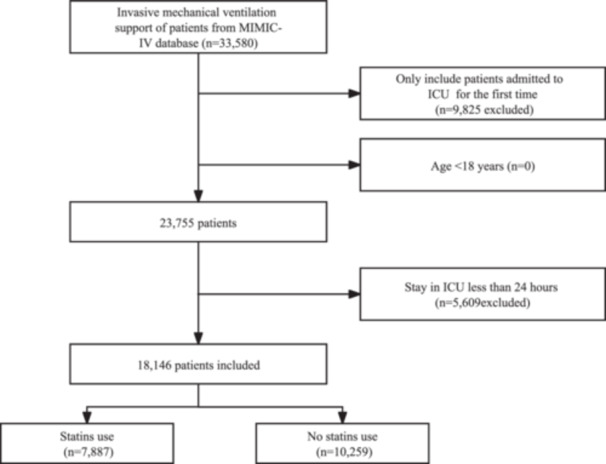
Study flowchart.

A total of 18,146 patients receiving invasive MV were included in our study, of whom 7887 (43.46%) were classified as “statin users” (Figure 1). The mean age of the entire study was 63.4 ± 16.0 years, and 39.8% of patients were women. The prevalence of delirium among all patients was 7.6% (1381/18,146). For patients who did not use a statin, the prevalence of delirium was 7.4% (757/10,259). The prevalence of delirium among those taking a statin was 7.9% (624/7887).

Table 1 illustrates the characteristics of the patient at baseline. Comparison of the two data sets indicated notable disparities. The group not using statins exhibited: a tendency towards younger age; a higher proportion of female patients; a higher prevalence of CRRT; a higher frequency of comorbid sepsis; longer duration of MV; longer DoIS and DoHS; significantly increased mortality rate at 30 and 90 days.

**Table 1:** Characteristics of study participants at baseline.

Variable	Total (*n* = 18,146)	No statin use (*n* = 10,259)	Statin use (*n* = 7887)	*p*
Age, year	63.4 ± 16.0	59.0 ± 17.6	69.1 ± 11.4	< 0.001
Sex, female, *n* (%)	7217 (39.8)	4468 (43.6)	2749 (34.9)	< 0.001
BMI, kg/m^2^	29.1 ± 6.6	28.7 ± 6.7	29.6 ± 6.4	< 0.001
Insurance type, *n* (%)				< 0.001
Medicaid	1377 (7.6)	1050 (10.2)	327 (4.1)	
Medicare	7920 (43.6)	3961 (38.6)	3959 (50.2)	
Other	8849 (48.8)	5248 (51.2)	3601 (45.7)	
Marital status, *n* (%)				< 0.001
Unmarried	7801 (43.0)	4879 (47.6)	2922 (37)	
Married	8277 (45.6)	4014 (39.1)	4263 (54.1)	
Unknown	2068 (11.4)	1366 (13.3)	702 (8.9)	
Ethnicity, *n* (%)				< 0.001
Caucasian	11,714 (64.6)	6254 (61)	5460 (69.2)	
Other	6432 (35.4)	4005 (39)	2427 (30.8)	
CRRT, *n* (%)	1354 (7.5)	913 (8.9)	441 (5.6)	< 0.001
Vasoactive drugs, *n* (%)	11,792 (65.0)	6003 (58.5)	5789 (73.4)	< 0.001
Heart rate (bpm)	85.7 ± 15.3	88.1 ± 16.6	82.6 ± 12.9	< 0.001
MAP (mmHg)	77.0 ± 9.6	77.9 ± 10.2	75.8 ± 8.5	< 0.001
Respiration rate (bpm)	19.1 ± 3.8	19.5 ± 4.1	18.5 ± 3.3	< 0.001
Temperature (°C)	36.9 ± 0.6	37.0 ± 0.7	36.8 ± 0.5	< 0.001
WBC count (×10^9^)	13.3 ± 6.8	13.2 ± 7.4	13.5 ± 6.0	0.012
Hb (g/L)	10.6 ± 2.0	10.7 ± 2.1	10.5 ± 1.8	< 0.001
Plt (×10^9^)	197.5 ± 102.6	203.8 ± 112.6	189.2 ± 87.3	< 0.001
Glucose (mmol/L)	143.2 ± 44.6	142.9 ± 47.6	143.5 ± 40.4	0.405
Sodium (mmol/L)	138.7 ± 4.5	138.9 ± 5.0	138.5 ± 3.8	< 0.001
Potassium (mmol/L)	4.3 ± 0.6	4.2 ± 0.6	4.4 ± 0.6	< 0.001
BUN (mg/dL)	18.5 (13.0, 29.5)	19.0 (12.5, 31.0)	18.5 (14.0, 27.5)	0.13
Scr (mg/dL)	1.0 (0.8, 1.5)	1.0 (0.7, 1.5)	1.0 (0.8, 1.4)	0.02
PH	7.4 ± 0.1	7.4 ± 0.1	7.4 ± 0.1	< 0.001
PCO2 (mmHg)	42.1 ± 9.1	42.3 ± 10.0	41.9 ± 7.7	0.01
PaO2/FiO2	281.3 ± 119.4	284.5 ± 125.4	277.0 ± 111.1	< 0.001
Lactate	2.4 ± 1.7	2.6 ± 1.9	2.2 ± 1.4	< 0.001
SAPS II	41.7 ± 14.6	41.3 ± 15.8	42.2 ± 12.7	< 0.001
SOFA score	6.4 ± 3.5	6.6 ± 3.9	6.3 ± 3.0	< 0.001
Charlson comorbidity index	5.6 ± 2.9	5.1 ± 3.1	6.1 ± 2.4	< 0.001
Sepsis, *n* (%)	12,705 (70.0)	7593 (74)	5112 (64.8)	< 0.001
MI, *n* (%)	3520 (19.4)	949 (9.3)	2571 (32.6)	< 0.001
CHF, *n* (%)	5040 (27.8)	2244 (21.9)	2796 (35.5)	< 0.001
CBVD, *n* (%)	3013 (16.6)	1642 (16)	1371 (17.4)	0.013
CPD, *n* (%)	4644 (25.6)	2465 (24)	2179 (27.6)	< 0.001
Rheumatic disease, *n* (%)	573 (3.2)	323 (3.1)	250 (3.2)	0.935
Diabetes without complication, *n* (%)	4301 (23.7)	1869 (18.2)	2432 (30.8)	< 0.001
Diabetes with complications, *n* (%)	1709 (9.4)	704 (6.9)	1005 (12.7)	< 0.001
Renal disease, *n* (%)	3477 (19.2)	1629 (15.9)	1848 (23.4)	< 0.001
Malignant cancer, *n* (%)	1972 (10.9)	1420 (13.8)	552 (7)	< 0.001
Severe liver disease, *n* (%)	1148 (6.3)	1044 (10.2)	104 (1.3)	< 0.001
Analgesia, h, median (IQR)	62.0 (27.0, 147.0)	68.0 (26.0, 171.5)	56.0 (28.0, 120.0)	< 0.001
Sedation, h, median (IQR)	62.0 (27.2, 161.0)	78.0 (33.0, 196.0)	48.0 (23.0, 116.0)	< 0.001
Delirium, *n* (%)	1381 (7.6)	757 (7.4)	624 (7.9)	0.18
Ventilation, h, median (IQR)	22.0 (10.0, 64.0)	29.0 (13.0, 81.0)	16.0 (8.0, 42.0)	< 0.001
ICU stay, d	3.7 (2.0, 7.7)	4.1 (2.2, 8.8)	3.2 (1.7, 6.2)	< 0.001
Hospital stay, d	9.2 (5.7, 15.9)	9.8 (5.6, 17.9)	8.8 (5.9, 13.9)	< 0.001
30‐day mortality, *n* (%)	3051 (16.8)	2275 (22.2)	776 (9.8)	< 0.001
90‐day mortality, *n* (%)	3264 (18.0)	2437 (23.8)	827 (10.5)	< 0.001

*Note:* For each variable, mean ± standard deviation, median (interquartile range), or number (percent) was reported (as appropriate).

Abbreviations: BMI, body mass index; BUN, blood urea nitrogen; CBVD, cerebrovascular disease; CHF, congestive heart failure; CPD, chronic pulmonary disease; CRRT, continuous renal replacement therapy; d, days; h, hours; Hb, hemoglobin; MAP, mean arterial pressure; MI, myocardial infarct; PLT, platelets; SAPS, simplified acute physiology Score; Scr, serum creatinine; SOFA, sequential organ failure assessment; WBC, white blood cell.

### 3.2 Outcomes

During univariate and multivariate logistic regression analyses, a considerably higher prevalence of delirium was observed in patients using statins. The univariate logistic regression analysis yielded an odds ratio (OR) of 1.08 (95% confidence interval [CI] = 0.97–1.2). In more comprehensive multivariate logistic regression analyses, the ORs for statin use exhibited consistent significance across all models (OR = 1.15–1.17, *p* < 0.05 for all). After adjustment for all the covariates listed in Table 2, an increased prevalence of delirium of 15% was evident in patients using statins (OR = 1.15, 95% CI = 1.01–1.37, *p* < 0.05, in model 6).

**Table 2:** Odds ratios and 95% confidence intervals of statin use for delirium.

	OR	95% CI	p
Model 1	1.08	(0.97~1.2)	0.18
Model 2	1.15	(1.02~1.3)	0.024
Model 3	1.15	(1.02~1.3)	0.027
Model 4	1.16	(1.03~1.31)	0.016
Model 5	1.17	(1.03~1.32)	0.015
Model 6	1.15	(1.01~1.31)	0.029
PSM	1.17	(1.01~1.37)	0.043

Abbreviations: CI, confidence interval; OR, odds ratio; PSM, propensity score matching.

Model 1: No adjustment.

Model 2: Age, sex, BMI, sedation duration, analgesia duration, SAPS II, SOFA score, sepsis, Charlson comorbidity index, pH, PCO_2_, PO_2_:FiO_2_ ratio, lactate.

Model 3: Model 2 plus WBC count, Hb, PLT, Scr, BUN, glucose, sodium, potassium.

Model 4: Model 3 plus body temperature, heart rate, MBP, respiration rate.

Model 5: Model 4 plus ICU duration, ventilation duration, CRRT, hospital stay, vasoactive drugs, insurance, marital status, ethnicity.

Model 6: Model 5 plus myocardial infarct, congestive heart failure, cerebrovascular disease, chronic pulmonary disease, renal disease, malignant cancer, rheumatic disease, diabetes mellitus without complication, diabetes with complication.

Inclusion of all the covariates listed in Tables 3 and 4 revealed that using statins did not have a significant impact on the DoIS (*β* = −0.09, 95% CI = −0.22 to 0.03). However, it was associated with a longer DoHS (*β* = 0.45, 95% CI = 0.13–0.76).

**Table 3:** Statin use and stay in the ICU (days).

		Model 1		Model 2	
Variable	*n*. total	*β* (95% CI)	*p*	*β* (95% CI)	*p*
No statin	10,259	0 (Ref)		0 (Ref)	
Statins	7887	−1.47 (−1.69~−1.26)	< 0.001	−0.09 (−0.22~0.03)	0.157

**Table 4:** Statin use and stay in hospital (days).

		Model 1		Model 2	
Variable	*n*. total	*β* (95% CI)	*p*	*β* (95% CI)	*p*
No statin	10,259	0 (Ref)		0 (Ref)	
Statins	7,887	−2.1 (−2.47~−1.73)	< 0.001	0.45 (0.13~0.76)	0.005

### 3.3 Subgroup Analysis and Sensitivity Analysis

**Figure 2:**
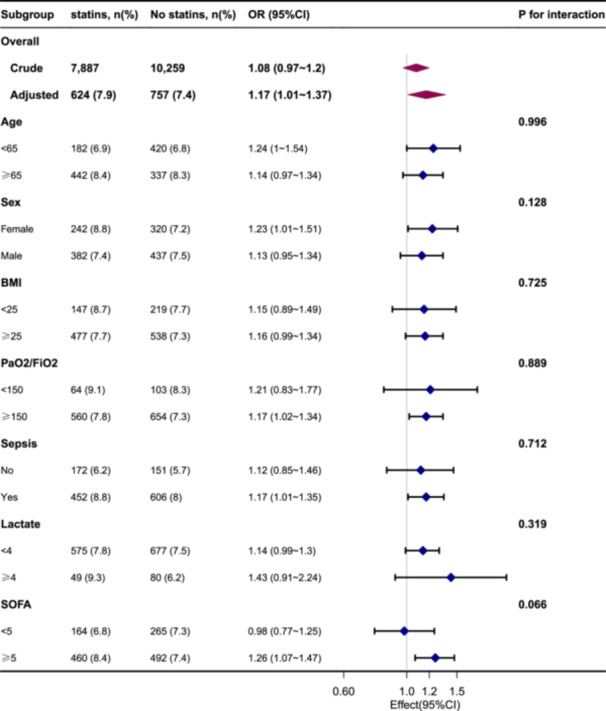
Association between statin use and delirium according to patient characteristics at baseline. Each stratification was adjusted for all factors, except the stratification factor itself. Abbreviations: BMI, body mass index; PaO_2_:FiO_2_, ratio of arterial partial pressure of oxygen‐to‐fraction of inspired oxygen; SOFA, sequential organ failure assessment.

Subgroup analyses indicated significant correlations between the use of statins and delirium in certain subgroups: young patients (age < 65 years); women; individuals with a high PaO2:FiO2 ratio; people with a high SOFA score; individuals with comorbidities (e.g., sepsis). Notably, the observed associations did not exhibit any significant interactions (Figure 2).

We also examined the effect of different types of statins on delirium. Patients were divided into five groups: those not taking statins; people using atorvastatin, pravastatin, or simvastatin; individuals using another type of statin. A higher probability of delirium in patients using statins was noted (OR = 1.13–1.26) (Table 5). We employed PSM to mitigate the potential influence of confounding variables on the association between statin use and delirium occurrence. Following PSM, 4811 well‐matched pairs were achieved for each group (Supporting Information S1: Table 1S). Within these PSM pairs, a significant increase in delirium prevalence was observed in the group using statins (401 (8.4%) vs. 347 (7.2%), *p* = 0.04). The multivariate logistic regression model indicated an OR of 1.17 (95% CI = 1.01–1.37, *p* < 0.05) for delirium prevalence (Table 2).

**Table 5:** Classification according to statin. statin use, and delirium risk.

			Model 1		Model 2	
Variable	*n*. total	*n.* event%	OR (95% CI)	*p*	OR (95% CI)	*p*
No statin	10,259	757 (7.4)	1 (Ref)		1 (Ref)	
Atorvastatin	4673	364 (7.8)	1.06 (0.93~1.21)	0.377	1.13 (0.98~1.32)	0.1
Pravastatin	572	49 (8.6)	1.18 (0.87~1.59)	0.293	1.24 (0.9~1.69)	0.183
Simvastatin	1997	156 (7.8)	1.06 (0.89~1.27)	0.5	1.14 (0.95~1.38)	0.166
Other	645	55 (8.5)	1.17 (0.88~1.56)	0.282	1.26 (0.93~1.7)	0.133

Model 1: No adjustment.

Model 2: Adjustment for age, sex, BMI, sedation duration, analgesia duration, SAPS II, SOFA score, sepsis, Charlson comorbidity index, pH, PCO_2_, PO_2_:FiO_2_ ratio, lactate, WBC count, Hb, PLT, Scr, BUN, glucose, sodium, potassium, temperature, heart rate, MBP, respiration rate, ICU stay, ventilation duration, CRRT, hospital stay, vasoactive drugs, insurance, marital status, ethnicity, myocardial infarct, congestive heart failure, cerebrovascular disease, chronic pulmonary disease, renal disease, malignant cancer, rheumatic disease, diabetes mellitus without complication, diabetes mellitus with complication.

## 4 Discussion

### 4.1 Main Findings

We conducted a cross‐sectional study to investigate the effect of statin use on the incidence of delirium in patients undergoing MV in the ICU. Our analysis revealed a substantial link between statin use in the ICU and an increased prevalence of delirium. Furthermore, statin administration to patients receiving MV in the ICU was associated with a reduced risk of delirium compared with patients receiving MV in the ICU but not taking statins. Notably, these results were consistent even after controlling for potential confounders using PSM.

### 4.2 Effects of Statin Use on Delirium and DoIS for Patients receiving MV

Univariate and multivariate logistic regression analyses revealed a significant rise in the prevalence of delirium among patients receiving MV and statins. Specifically, a higher prevalence of 15% was observed in statin users (OR = 1.15, 95% CI = 1.01–1.37, *p* < 0.05, model 6). These results are consistent with data from earlier investigations, in which researchers observed a higher prevalence of delirium in individuals using statins. They hypothesized that this could be attributed to the release of nitric oxide induced by statins, resulting in microvascular dilation, shunting, and subsequent cerebral ischemia [30]. Redelmeier et al. evaluated individuals aged ≥ 65 years undergoing elective surgery and found statin utilization in this cohort to be linked to a higher probability of developing postoperative delirium [31]. Mather et al. adopted a retrospective cohort design and did not find an association between statin use and delirium occurrence. Their study encompassed a substantial sample size, and they controlled meticulously for potential confounders, which lent credibility to their results. Their study involved 1475 patients admitted to the medical ICU, of whom half received statin treatment throughout their hospitalization, whereas the other half did not. Utilizing a Cox proportional hazards model, they found that the likelihood of experiencing delirium during hospitalization was reduced by 50% in the statin‐treated group (OR = 0.47, 95% CI = 0.38–0.56) [32]. Their study had limitations because they did not consider the competing risks associated with delirium (e.g., coma) or variables that could influence delirium onset (e.g., illness severity, exposure to benzodiazepines), which can vary each day [33]. In one investigation, daily utilization of statins before hospitalization and throughout their ICU admission was monitored for 763 patients [1]. Statin utilization in the ICU was associated with a reduction in delirium occurrence. Among patients with sepsis, statin administration correlated with a reduced likelihood of developing delirium upon ICU admission. Similarly, patients not suffering from sepsis receiving statin therapy showed a lower likelihood of experiencing delirium as their ICU stay progressed. A recent meta‐analysis involving six observational studies revealed no significant correlation between statin use and delirium occurrence [34]. However, it is worth noting that 98% of the combined cohort comprised patients who underwent elective surgery, indicating a minimal risk of delirium [31].

Our study indicated that statin utilization did not impact the DoIS, a notable contributing factor to delirium onset [35, 36]. The increased prevalence of delirium in ICU patients receiving MV due to statin administration remained unaffected even after categorizing statins individually and conducting PSM. These results confirmed that statin utilization in ICU patients receiving MV increased the prevalence of delirium markedly. Furthermore, statin use may have lengthened the DoHS.

### 4.3 Strengths of Our Study

Our study had four key strengths. First, specific exploration of the effects of statin use on delirium risk in patients receiving MV has not been done before. Our results offer proof that statin use heightens the likelihood of delirium in patients receiving MV. Second, our findings are pertinent in patients undergoing MV due to the extensive use of statins in the primary and secondary prevention of cardiovascular disease. Third, we employed multiple regression analysis and conducted PSM to bolster the reliability and robustness of our study outcomes. Fourth, delirium is a multifactorial condition that is challenging to reverse, but our findings may have clinical relevance in identifying at‐risk individuals and preventing delirium.

### 4.4 Limitations of Our Study

Our research had three main limitations. First, as with all retrospective analyses, the possibility of residual confounders (e.g., duration of tobacco smoking and alcohol utilization) cannot be ignored. We addressed potential confounding variables and reduced the impact of factors that could induce outcome bias using PSM. Second, caution should be exercised when generalizing our findings because our sample was limited to a single ICU institution and a single country (USA). Prospective studies involving multiple centers could verify our outcomes. Third, MV is a critical contributor to delirium, but it is not the sole factor. Our study population consisted of patients who were admitted to the ICU and received invasive MV, and thus, the results are applicable only to this group. Nonetheless, MV contributed significantly to the prevalence of delirium, and we accounted for several other crucial factors through covariate adjustments. It is also unclear whether there is a correlation between the use of statins and the occurrence of delirium. Fourth, the MIMIC database system contains a substantial amount of missing data regarding patients' statin dose, duration, and indication information. Owing to these data limitations, we were unable to comprehensively integrate these factors into the current analyses. Fifth, there is insufficient data on how long patients have been on statin therapy before admission to the ICU. Therefore, no comparison could be made between duration of statin use and delirium results. Sixth, this study lacks the systematic monitoring of patients' sedation levels and consciousness levels using standardized tools such as the Glasgow Coma Scale (GCS). Due to the retrospective nature of our research, the medical records had inconsistent documentation regarding GCS scores and detailed sedation information. This aspect of the trial will be subject to further refinement in subsequent trials.

## 5 Conclusion

The findings of this retrospective cross‐sectional study indicate that statin use is linked to an increased risk of delirium in ICU patients receiving MV. Additional research is necessary to validate this association.

## Author Contributions

**Zhi Liu:** writing – original draft. **Zhe Li:** conceptualization and methodology. **Qihai Wan:** software, formal analysis, and project administration. **Yi Yu:** writing – review and editing, visualization, validation, supervision, and data curation.

## Ethics Statement

The research, which involved human participants, was thoroughly reviewed and received ethical approval from both the Massachusetts Institute of Technology and the Beth Israel Deaconess Medical Center.

## Conflicts of Interest

The authors declare no conflicts of interest.

## Transparency Statement

The corresponding author, Yi Yu, affirms that this manuscript is an honest, accurate, and transparent account of the study being reported; that no important aspects of the study have been omitted; and that any discrepancies from the study as planned (and, if relevant, registered) have been explained.

## Supporting information

Table 1s.

## Data Availability

This study utilized data from the MIMIC‐IV clinical database, which is subject to strict licensing requirements and usage restrictions. Access to the MIMIC‐IV database requires user verification, completion of relevant training programs (e.g., CITI Data or Specimens Only Research), and approval of a project‐specific data use agreement. These measures ensure the responsible and compliant use of the database in accordance with applicable regulations and ethical standards.

## References

[1] A. Morandi , C. G. Hughes , J. L. Thompson , et al., “Statins and Delirium During Critical Illness: A Multicenter, Prospective Cohort Study,” Critical Care Medicine 42, no. 8 (2014): 1899–1909. 24810528 10.1097/CCM.0000000000000398 PMC4103957

[2] P. P. Pandharipande , T. D. Girard , J. C. Jackson , et al., “Long‐Term Cognitive Impairment After Critical Illness,” New England Journal of Medicine 369, no. 14 (2013): 1306–1316. 24088092 10.1056/NEJMoa1301372 PMC3922401

[3] J. T. Denney and J. D. Boardman , “Hearing Impairment, Household Composition, Marital Status, and Mortality Among U.S. Adults,” Journals of Gerontology: Series B 76, no. 1 (2021): 201–208. 10.1093/geronb/gbz157 31814013

[4] K. Sosnowski , F. Lin , W. Chaboyer , K. Ranse , A. Heffernan , and M. Mitchell , “The Effect of the ABCDE/ABCDEF Bundle on Delirium, Functional Outcomes, and Quality of Life in Critically Ill Patients: A Systematic Review and Meta‐Analysis,” International Journal of Nursing Studies 138 (2023): 104410. 36577261 10.1016/j.ijnurstu.2022.104410

[5] C. Dubiel , B. M. Hiebert , A. N. Stammers , et al., “Delirium Definition Influences Prediction of Functional Survival in Patients One‐Year Postcardiac Surgery,” Journal of Thoracic and Cardiovascular Surgery 163, no. 2 (2022): 725–734. 32859411 10.1016/j.jtcvs.2020.07.028

[6] J. Barr , G. L. Fraser , K. Puntillo , et al., “Clinical Practice Guidelines for the Management of Pain, Agitation, and Delirium in Adult Patients in the Intensive Care Unit,” Critical Care Medicine 41, no. 1 (2013): 263–306. 23269131 10.1097/CCM.0b013e3182783b72

[7] J. E. Wilson , M. F. Mart , C. Cunningham , et al., “Delirium,” Nature Reviews Disease Primers 6, no. 1 (2020): 90. 10.1038/s41572-020-00223-4 PMC9012267 33184265

[8] J. R. Maldonado , “Neuropathogenesis of Delirium: Review of Current Etiologic Theories and Common Pathways,” American Journal of Geriatric Psychiatry 21, no. 12 (2013): 1190–1222. 10.1016/j.jagp.2013.09.005 24206937

[9] K. Rump and M. Adamzik , “Epigenetic Mechanisms of Postoperative Cognitive Impairment Induced by Anesthesia and Neuroinflammation,” Cells 11, no. 19 (2022): 2954. 36230916 10.3390/cells11192954 PMC9563723

[10] X. P. Wang , D. Lv , Y. F. Chen , et al., “Impact of Pain, Agitation, and Delirium Bundle on Delirium and Cognitive Function,” Journal of Nursing Research 30, no. 4 (2022): e222. 10.1097/jnr.0000000000000497 35608396

[11] N. Inagaki‐Katashiba , T. Ito , M. Inaba , et al., “Statins Can Suppress DC‐Mediated Th2 Responses Through the Repression of OX40‐Ligand and CCL17 Expression,” European Journal of Immunology 49, no. 11 (2019): 2051–2062. 31269241 10.1002/eji.201847992 PMC6899642

[12] X. B. Yu , H. N. Zhang , Y. Dai , et al., “Simvastatin Prevents and Ameliorates Depressive Behaviors via Neuroinflammatory Regulation in Mice,” Journal of Affective Disorders 245 (2019): 939–949. 30699879 10.1016/j.jad.2018.11.086

[13] A. L. Sørensen , H. C. Hasselbalch , C. H. Nielsen , H. E. Poulsen , and C. Ellervik , “Statin Treatment, Oxidative Stress and Inflammation in a Danish Population,” Redox Biology 21 (2019): 101088. 30594900 10.1016/j.redox.2018.101088 PMC6307042

[14] J. W. Devlin , Y. Skrobik , C. Gélinas , et al., “Clinical Practice Guidelines for the Prevention and Management of Pain, Agitation/Sedation, Delirium, Immobility, and Sleep Disruption in Adult Patients in the ICU,” Critical Care Medicine 46, no. 9 (2018): e825–e873. 30113379 10.1097/CCM.0000000000003299

[15] L. W. Chen , C. S. Lin , M. C. Tsai , et al., “Pitavastatin Exerts Potent Anti‐Inflammatory and Immunomodulatory Effects via the Suppression of AP‐1 Signal Transduction in Human T Cells,” International Journal of Molecular Sciences 20, no. 14 (2019): 3534. 31330988 10.3390/ijms20143534 PMC6678418

[16] J. H. Chen , T. Wu , L. K. Yang , et al., “Protective Effects of Atorvastatin on Cerebral Vessel Autoregulation in an Experimental Rabbit Model of Subarachnoid Hemorrhage,” Molecular Medicine Reports 17, no. 1 (2018): 1651–1659. 29257200 10.3892/mmr.2017.8074 PMC5780106

[17] N. Zhang , C. Song , B. Zhao , et al., “Neovascularization and Synaptic Function Regulation With Memantine and Rosuvastatin in a Rat Model of Chronic Cerebral Hypoperfusion,” Journal of Molecular Neuroscience 63, no. 2 (2017): 223–232. 28920182 10.1007/s12031-017-0974-1

[18] G. Mariscalco , M. Cottini , M. Zanobini , et al., “Preoperative Statin Therapy Is not Associated With a Decrease in the Incidence of Delirium After Cardiac Operations,” Annals of Thoracic Surgery 93, no. 5 (2012): 1439–1447. 22541176 10.1016/j.athoracsur.2012.02.012

[19] T. K. Oh , H. Y. Park , H. J. Shin , Y. T. Jeon , S. H. Do , and J. W. Hwang , “The Role of Perioperative Statin Use in the Prevention of Delirium After Total Knee Replacement Under Spinal Anesthesia,” Journal of Arthroplasty 33, no. 12 (2018): 3666–3671 e1. 30236494 10.1016/j.arth.2018.08.022

[20] V. J. Page , D. Davis , X. B. Zhao , et al., “Statin Use and Risk of Delirium in the Critically Ill,” American Journal of Respiratory and Critical Care Medicine 189, no. 6 (2014): 666–673. 24417431 10.1164/rccm.201306-1150OC PMC3974585

[21] J. Y. An , J. Y. Park , J. Cho , H. E. Kim , J. Park , and J. Oh , “The Relationship Between Delirium and Statin Use According to Disease Severity in Patients in the Intensive Care Unit,” Clinical Psychopharmacology and Neuroscience 21, no. 1 (2023): 179–187. 36700324 10.9758/cpn.2023.21.1.179 PMC9889904

[22] J. Xia , C. Hu , L. Wang , and Y. Zhang , “Association Between Statin Use on Delirium and 30‐Day Mortality in Patients With Chronic Obstructive Pulmonary Disease in the Intensive Care Unit,” European Journal of Medical Research 28, no. 1 (2023): 572. 38062497 10.1186/s40001-023-01551-3 PMC10704755

[23] A. E. W. Johnson , L. Bulgarelli , L. Shen , et al., “MIMIC‐IV, a Freely Accessible Electronic Health Record Dataset,” Scientific Data 10, no. 1 (2023): 1. 36596836 10.1038/s41597-022-01899-x PMC9810617

[24] E. Elm , D. G. Altman , M. Egger , S. J. Pocock , P. C. Gøtzsche , and J. P. Vandenbroucke , “Strengthening the Reporting of Observational Studies in Epidemiology (STROBE) Statement: Guidelines for Reporting Observational Studies,” BMJ 335, no. 7624 (2007): 806–808. 17947786 10.1136/bmj.39335.541782.AD PMC2034723

[25] D. Gusmao‐Flores , J. I. F. Salluh , R. Á. Chalhub , and L. C. Quarantini , “The Confusion Assessment Method for the Intensive Care Unit (CAM‐ICU) and Intensive Care Delirium Screening Checklist (ICDSC) for the Diagnosis of Delirium: A Systematic Review and Meta‐Analysis of Clinical Studies,” Critical Care 16, no. 4 (2012): R115. 22759376 10.1186/cc11407 PMC3580690

[26] Ö. E. Dallı , Y. Yıldırım , F. Ş. Aykar , and F. Kahveci , “The Effect of Music on Delirium, Pain, Sedation and Anxiety in Patients Receiving Mechanical Ventilation in the Intensive Care Unit,” Intensive and Critical Care Nursing 75 (2023): 103348. 36470699 10.1016/j.iccn.2022.103348

[27] K. Heybati , F. Zhou , S. Ali , et al., “Outcomes of Dexmedetomidine Versus Propofol Sedation in Critically Ill Adults Requiring Mechanical Ventilation: A Systematic Review and Meta‐Analysis of Randomised Controlled Trials,” British Journal of Anaesthesia 129, no. 4 (2022): 515–526. 35961815 10.1016/j.bja.2022.06.020

[28] K. Lewis , F. Alshamsi , K. L. Carayannopoulos , et al., “Dexmedetomidine vs Other Sedatives in Critically Ill Mechanically Ventilated Adults: A Systematic Review and Meta‐Analysis of Randomized Trials,” Intensive Care Medicine 48, no. 7 (2022): 811–840. 35648198 10.1007/s00134-022-06712-2

[29] E. A. Álvarez , M. A. Garrido , E. A. Tobar , et al., “Occupational Therapy for Delirium Management in Elderly Patients Without Mechanical Ventilation in an Intensive Care Unit: A Pilot Randomized Clinical Trial,” Journal of Critical Care 37 (2017): 85–90. 27660922 10.1016/j.jcrc.2016.09.002

[30] F. Ma and Z. C. Han , “Statins, Nitric Oxide and Neovascularization,” Cardiovascular Drug Reviews 23, no. 4 (2005): 281–292. 16614729 10.1111/j.1527-3466.2005.tb00173.x

[31] D. A. Redelmeier , D. Thiruchelvam , and N. Daneman , “Delirium After Elective Surgery Among Elderly Patients Taking Statins,” Canadian Medical Association Journal 179, no. 7 (2008): 645–652. 18809895 10.1503/cmaj.080443 PMC2535740

[32] J. F. Mather , J. P. Corradi , C. Waszynski , et al., “Statin and Its Association With Delirium in the Medical ICU,” Critical Care Medicine 45, no. 9 (2017): 1515–1522. 28622167 10.1097/CCM.0000000000002530

[33] I. J. Zaal , J. W. Devlin , M. Hazelbag , et al., “Benzodiazepine‐Associated Delirium in Critically Ill Adults,” Intensive Care Medicine 41, no. 12 (2015): 2130–2137. 26404392 10.1007/s00134-015-4063-z

[34] Y. H. Chang , J. Y. Wang , T. R. Peng , J. H. Lian , M. C. Lee , and H. M. Chen , “Statin Use and Delirium Risk: An Updated Systematic Review and Meta‐Analysis,” American Journal of Therapeutics 30, no. 4 (2023): e326–e335. 36728521 10.1097/MJT.0000000000001593

[35] H. Chen , L. Mo , H. Hu , Y. Ou , and J. Luo , “Risk Factors of Postoperative Delirium After Cardiac Surgery: A Meta‐Analysis,” Journal of Cardiothoracic Surgery 16, no. 1 (2021): 113. 33902644 10.1186/s13019-021-01496-w PMC8072735

[36] A. Cortés‐Beringola , L. Vicent , R. Martín‐Asenjo , et al., “Diagnosis, Prevention, and Management of Delirium in the Intensive Cardiac Care Unit,” American Heart Journal 232 (2021): 164–176. 33253676 10.1016/j.ahj.2020.11.011

