# Supplementary material for: Association Between Statin Use in the Intensive Care Unit and Delirium in Patients Receiving Mechanical Ventilation: A Cross‐Sectional Study
Source: Health Sci Rep. 2025 Jul 21;8(7):e71013. doi: 10.1002/hsr2.71013 (PMC12280229; doi:10.1002/hsr2.71013)
Supplement: Supplementary file 1 — Table 1s. [file HSR2-8-e71013-s001.docx]

| **Variable** | **Unmatched patients** | | | | **Patients matched by propensity score** | | | |
| --- | --- | --- | --- | --- | --- | --- | --- | --- |
|  | **Total**  **(n = 18,146)** | **No statin use**  **(n = 10,259)** | **Statin use**  **(n = 7,887)** | **P** | **Total (n = 9,622)** | **No statin use**  **(n = 4,811)** | **Statin use**  **(n = 4,811)** | **P** |
| Age, year | 63.4 ± 16.0 | 59.0 ± 17.6 | 69.1 ± 11.4 | < 0.001 | 66.3 ± 14.1 | 65.6 ± 15.8 | 67.1 ± 12.1 | < 0.001 |
| Sex, female, n (%) | 7,217 (39.8) | 4,468 (43.6) | 2,749 (34.9) | < 0.001 | 3779 (39.3) | 1946 (40.4) | 1833 (38.1) | 0.018 |
| BMI, kg/m^2^ | 29.1 ± 6.6 | 28.7 ± 6.7 | 29.6 ± 6.4 | < 0.001 | 29.4 ± 6.6 | 29.1 ± 6.6 | 29.7 ± 6.6 | < 0.001 |
| Insurance type, n (%) |  |  |  | < 0.001 |  |  |  | 0.043 |
| Medicaid™ | 1,377 ( 7.6) | 1,050 (10.2) | 327 (4.1) |  | 516 ( 5.4) | 255 (5.3) | 261 (5.4) |  |
| Medicare™ | 7,920 (43.6) | 3,961 (38.6) | 3,959 (50.2) |  | 4592 (47.7) | 2357 (49) | 2235 (46.5) |  |
| other | 8,849 (48.8) | 5,248 (51.2) | 3,601 (45.7) |  | 4514 (46.9) | 2199 (45.7) | 2315 (48.1) |  |
| Marital status, n (%) |  |  |  | < 0.001 |  |  |  | 0.339 |
| unmarried | 7,801 (43.0) | 4,879 (47.6) | 2,922 (37) |  | 3969 (41.2) | 2010 (41.8) | 1959 (40.7) |  |
| married | 8,277 (45.6) | 4,014 (39.1) | 4,263 (54.1) |  | 4671 (48.5) | 2329 (48.4) | 2342 (48.7) |  |
| unknown | 2,068 (11.4) | 1,366 (13.3) | 702 (8.9) |  | 982 (10.2) | 472 (9.8) | 510 (10.6) |  |
| Ethnicity, n (%) |  |  |  | < 0.001 |  |  |  | 0.698 |
| Caucasian | 11,714 (64.6) | 6,254 (61) | 5,460 (69.2) |  | 6394 (66.5) | 3188 (66.3) | 3206 (66.6) |  |
| other | 6,432 (35.4) | 4005 (39) | 2,427 (30.8) |  | 3228 (33.5) | 1623 (33.7) | 1605 (33.4) |  |
| CRRT, n (%) | 1,354 ( 7.5) | 913 (8.9) | 441 (5.6) | < 0.001 | 670 ( 7.0) | 322 (6.7) | 348 (7.2) | 0.298 |
| Vasoactive drugs, n (%) | 11,792 (65.0) | 6,003 (58.5) | 5,789 (73.4) | < 0.001 | 6418 (66.7) | 3175 (66) | 3243 (67.4) | 0.141 |
| Heart rate (bpm) | 85.7 ± 15.3 | 88.1 ± 16.6 | 82.6 ± 12.9 | < 0.001 | 83.8 ± 14.4 | 83.5 ± 15.0 | 84.1 ± 13.9 | 0.082 |
| MAP (mmHg) | 77.0 ± 9.6 | 77.9 ± 10.2 | 75.8 ± 8.5 | < 0.001 | 76.6 ± 9.2 | 76.7 ± 9.5 | 76.5 ± 9.0 | 0.506 |
| Respiration rate (bpm) | 19.1 ± 3.8 | 19.5 ± 4.1 | 18.5 ± 3.3 | < 0.001 | 19.0 ± 3.5 | 18.9 ± 3.6 | 19.1 ± 3.5 | < 0.001 |
| SPO_2_ (%) | 97.6 ± 2.2 | 97.5 ± 2.4 | 97.6 ± 1.8 | 0.004 | 97.6 ± 2.2 | 97.6 ± 2.4 | 97.5 ± 2.0 | 0.317 |
| Temperature (℃) | 36.9 ± 0.6 | 37.0 ± 0.7 | 36.8 ± 0.5 | < 0.001 | 36.9 ± 0.6 | 36.9 ± 0.6 | 36.9 ± 0.6 | 0.745 |
| WBC count (×10^9^ ) | 13.3 ± 6.8 | 13.2 ± 7.4 | 13.5 ± 6.0 | 0.012 | 13.5 ± 6.9 | 13.3 ± 7.5 | 13.8 ± 6.2 | < 0.001 |
| Hb (g/L) | 10.6 ± 2.0 | 10.7 ± 2.1 | 10.5 ± 1.8 | < 0.001 | 10.6 ± 1.9 | 10.6 ± 2.0 | 10.7 ± 1.9 | 0.074 |
| Plt (×10^9^) | 197.5 ± 102.6 | 203.8 ± 112.6 | 189.2 ± 87.3 | < 0.001 | 198.8 ± 96.5 | 198.9 ± 98.1 | 198.7 ± 95.0 | 0.926 |
| Glucose (mmol/L) | 143.2 ± 44.6 | 142.9 ± 47.6 | 143.5 ± 40.4 | 0.405 | 146.2 ± 45.3 | 144.9 ± 45.6 | 147.4 ± 44.9 | 0.007 |
| Sodium (mmol/L) | 138.7 ± 4.5 | 138.9 ± 5.0 | 138.5 ± 3.8 | < 0.001 | 138.7 ± 4.3 | 138.7 ± 4.6 | 138.7 ± 4.1 | 0.439 |
| Potassium (mmol/L) | 4.3 ± 0.6 | 4.2 ± 0.6 | 4.4 ± 0.6 | < 0.001 | 4.3 ± 0.6 | 4.3 ± 0.6 | 4.3 ± 0.6 | < 0.001 |
| BUN (mg/dL) | 18.5 (13.0, 29.5) | 19.0 (12.5, 31.0) | 18.5 (14.0, 27.5) | 0.13 | 19.5 (14.0, 30.5) | 19.5 (13.5, 31.0) | 19.5 (14.0, 30.0) | 0.306 |
| Scr (mg/dL) | 1.0 (0.8, 1.5) | 1.0 (0.7, 1.5) | 1.0 (0.8, 1.4) | 0.02 | 1.0 (0.8, 1.5) | 1.0 (0.8, 1.5) | 1.0 (0.8, 1.5) | 0.001 |
| pH | 7.4 ± 0.1 | 7.4 ± 0.1 | 7.4 ± 0.1 | < 0.001 | 7.4 ± 0.1 | 7.4 ± 0.1 | 7.3 ± 0.1 | < 0.001 |
| PO_2_ (mmHg) | 194.9 ± 82.5 | 181.7 ± 84.5 | 212.0 ± 76.4 | < 0.001 | 196.6 ± 84.0 | 198.1 ± 89.7 | 195.1 ± 77.8 | 0.082 |
| PCO_2_ (mmHg) | 42.1 ± 9.1 | 42.3 ± 10.0 | 41.9 ± 7.7 | 0.01 | 42.8 ± 9.2 | 42.3 ± 9.6 | 43.2 ± 8.9 | < 0.001 |
| PaO_2_:FiO_2_ ratio | 281.3 ± 119.4 | 284.5 ± 125.4 | 277.0 ± 111.1 | < 0.001 | 279.0 ± 117.0 | 281.8 ± 115.2 | 276.1 ± 118.6 | 0.016 |
| Lactate | 2.4 ± 1.7 | 2.6 ± 1.9 | 2.2 ± 1.4 | < 0.001 | 2.3 ± 1.4 | 2.3 ± 1.3 | 2.4 ± 1.5 | 0.002 |
| SAPS II | 41.7 ± 14.6 | 41.3 ± 15.8 | 42.2 ± 12.7 | < 0.001 | 42.6 ± 14.2 | 42.3 ± 14.7 | 42.9 ± 13.7 | 0.024 |
| SOFA score | 6.4 ± 3.5 | 6.6 ± 3.9 | 6.3 ± 3.0 | < 0.001 | 6.4 ± 3.4 | 6.2 ± 3.5 | 6.5 ± 3.2 | < 0.001 |
| Charlson Comorbidity Index | 5.6 ± 2.9 | 5.1 ± 3.1 | 6.1 ± 2.4 | < 0.001 | 5.9 ± 2.8 | 5.9 ± 3.0 | 5.9 ± 2.6 | 0.95 |
| Sepsis, n (%) | 12705 (70.0) | 7593 (74) | 5112 (64.8) | < 0.001 | 6701 (69.6) | 3335 (69.3) | 3366 (70) | 0.492 |
| MI, n (%) | 3520 (19.4) | 949 (9.3) | 2571 (32.6) | < 0.001 | 1867 (19.4) | 832 (17.3) | 1035 (21.5) | < 0.001 |
| CHF, n (%) | 5040 (27.8) | 2244 (21.9) | 2796 (35.5) | < 0.001 | 3112 (32.3) | 1533 (31.9) | 1579 (32.8) | 0.316 |
| CBVD, n (%) | 3013 (16.6) | 1642 (16) | 1371 (17.4) | 0.013 | 1727 (17.9) | 887 (18.4) | 840 (17.5) | 0.212 |
| CPD, n (%) | 4644 (25.6) | 2465 (24) | 2179 (27.6) | < 0.001 | 2741 (28.5) | 1336 (27.8) | 1405 (29.2) | 0.119 |
| Rheumatic disease, n (%) | 573 ( 3.2) | 323 (3.1) | 250 (3.2) | 0.935 | 318 ( 3.3) | 165 (3.4) | 153 (3.2) | 0.494 |
| Diabetes without complication, n (%) | 4301 (23.7) | 1869 (18.2) | 2432 (30.8) | < 0.001 | 2559 (26.6) | 1274 (26.5) | 1285 (26.7) | 0.8 |
| Diabetes with complication, n (%) | 1709 ( 9.4) | 704 (6.9) | 1005 (12.7) | < 0.001 | 1052 (10.9) | 509 (10.6) | 543 (11.3) | 0.267 |
| Renal disease, n (%) | 3477 (19.2) | 1629 (15.9) | 1848 (23.4) | < 0.001 | 2135 (22.2) | 1045 (21.7) | 1090 (22.7) | 0.27 |
| Malignant cancer, n (%) | 1972 (10.9) | 1420 (13.8) | 552 (7) | < 0.001 | 935 ( 9.7) | 477 (9.9) | 458 (9.5) | 0.513 |
| Analgesia, h, median (IQR) | 62.0 (27.0, 147.0) | 68.0 (26.0, 171.5) | 56.0 (28.0, 120.0) | < 0.001 | 60.0 (27.0, 144.0) | 61.0 (26.0, 151.0) | 60.0 (28.0, 138.0) | 0.479 |
| Sedation, h, median (IQR) | 62.0 (27.2, 161.0) | 78.0 (33.0, 196.0) | 48.0 (23.0, 116.0) | < 0.001 | 62.0 (27.0, 156.0) | 65.0 (28.0, 162.0) | 60.0 (27.0, 151.0) | 0.061 |
| Delirium, n (%) | 1381 ( 7.6) | 757 (7.4) | 624 (7.9) | 0.18 | 748 ( 7.8) | 347 (7.2) | 401 (8.3) | 0.04 |
| Ventilation, h, median (IQR) | 22.0 (10.0, 64.0) | 29.0 (13.0, 81.0) | 16.0 (8.0, 42.0) | < 0.001 | 22.0 (10.0, 64.0) | 24.0 (12.0, 66.0) | 20.0 (9.0, 60.0) | < 0.001 |
| ICU stay, d | 3.7 (2.0, 7.7) | 4.1 (2.2, 8.8) | 3.2 (1.7, 6.2) | < 0.001 | 3.8 (2.0, 7.7) | 3.9 (2.1, 7.8) | 3.6 (1.9, 7.4) | < 0.001 |
| Hospital stay, d | 9.2 (5.7, 15.9) | 9.8 (5.6, 17.9) | 8.8 (5.9, 13.9) | < 0.001 | 9.0 (5.7, 15.7) | 9.0 (5.5, 16.1) | 9.1 (5.8, 15.2) | 0.036 |
| 30-day mortality, n (%) | 3051 (16.8) | 2275 (22.2) | 776 (9.8) | < 0.001 | 1647 (17.1) | 1047 (21.8) | 600 (12.5) | < 0.001 |
| 90-day mortality, n (%) | 3264 (18.0) | 2437 (23.8) | 827 (10.5) | < 0.001 | 1736 (18.0) | 1095 (22.8) | 641 (13.3) | < 0.001 |

**Table 1S:** Characteristics of study participants at baseline after PSM.

For each variable, mean ± standard deviation, median (interquartile range), or number (percent) was reported (as appropriate). PSM, Propensity-score matching.

h, hours; d, days; BMI, body mass index; MAP, mean arterial pressure; SPO_2_, pulse oxygen saturation; WBC, white blood cell; Hb, hemoglobin; PLT, platelets; BUN, blood urea nitrogen; Scr, serum creatinine; MI, myocardial infarct; CHF, congestive heart failure; CBVD, cerebrovascular disease; CPD, chronic pulmonary disease; SAPS, Simplified Acute Physiology Score; SOFA, Sequential Organ Failure Assessment; CRRT, continuous renal replacement therapy.
